# Formulation of a Culture Medium to Optimize the Production of Lipopeptide Biosurfactant by a New Isolate of *Bacillus* sp.: A Soil Heavy Metal Mitigation Approach

**DOI:** 10.3389/fmicb.2022.785985

**Published:** 2022-03-08

**Authors:** Sahar Kalvandi, Hamidreza Garousin, Ahmad Ali Pourbabaee, Hossein Ali Alikhani

**Affiliations:** Biology and Biotechnology Lab, Department of Soil Science, University College of Agriculture and Natural Resources, University of Tehran, Karaj, Iran

**Keywords:** optimization, response surface methodology (RSM), soil bioremediation, lipopeptide biosurfactant, heavy metals

## Abstract

This research aimed to optimize a lipopeptide biosurfactant produced from *Bacillus* sp. SHA302 due to its high efficiency of heavy metal release in soil. The results demonstrated that the metal release capacity of the lipopeptide biosurfactant alone increased with increasing the biosurfactant concentration. Among treatments with different biosurfactant concentrations plus acid, the highest metal release rates of 53.8% ± 1.4 and 39.3% ± 1.7 for Zn and Pb, respectively, were observed in the critical micelle concentration (CMC) + HCl treatment. The results of a factorial experiment designed for optimizing biosurfactant production showed that among five inexpensive carbon sources and six mineral nitrogen sources, sugar beet molasses (1%) and ammonium chloride (0.1%) were the most efficient sources in lowering the surface tension (ST) of the culture media to 32.2 ± 0.76 mN/m. The second step of the experiment was a Plackett–Burman design with 11 factors and showed that the four factors of pH, ammonium chloride, magnesium sulfate, and molasses significantly affected (*P* < 0.05) the changes in ST and biosurfactant production. The third step of the experiment was done using the response surface methodology (RSM) with a central composite design. The results showed that a pH of 7.3, 1.5 g/l of ammonium chloride, 0.3 g/l of magnesium sulfate, and 10% of sugar beet molasses yielded values of 29.2 ± 0.71 mN/m and 5.74 ± 0.52 g/l for the two variables of ST and biosurfactant production, respectively, which reached their most optimal levels.

## Introduction

Heavy metals are considered permanent soil pollutants, especially in industrial countries (Santos et al., 2016). These pollutants can pose a hazard to human health and ecosystems through the food chain or entry into the soil and water resources. Heavy metal pollution is a concern globally (Chakraborty and Das, 2014; Mao et al., 2015). A promising solution to achieve low bioavailability of pollutants is the use of biosurfactants that play an essential role in the desorption of pollutants from the surface of soil particles. Biosurfactants increase the presence of pollutants in the solution phase, thereby increasing their availability to microorganisms that can break them down (Mao et al., 2015). Microorganisms produce surfactants to increase the solubility, biodegradation, and bioavailability of pollutants in their environments (Rane et al., 2017). Surfactants are amphiphilic molecules with various structural and functional groups and wide-ranging characteristics such as reducing surface and interfacial tension formation of micelle and microemulsion between two different phases. These characteristics increase the availability of the pollutants and their subsequent biodegradation (Pacwa-Płociniczak et al., 2011; Soberón-Chávez and Maier, 2011). Heavy metals are usually adsorbed as ions or charged ionic pairs to soil surfaces. Unlike organic pollutants, these compounds enter the soil solution either by forming non-ionic complexes or through an ionic exchange (Ochoa-Loza et al., 2001; Swarnkar et al., 2012).

Although biosurfactants have many benefits, their expensive production and low production quantity hinder their use in industry (Moussa et al., 2014). An effective method to reduce limitations in biosurfactant production is to determine optimal levels of nutrients and ideal conditions of microbial cultures (Kim and Kim, 2013). To optimize culture media for biosurfactant production, the culture conditions (temperature, shaking rate, pH, etc.) and media components (sources of nitrogen, carbon, micronutrients, etc.) should be identified and optimized among other constituents involved in the production (Singh et al., 2016). For industrial-scale biosurfactant production, the feedstock value can reach 50% of all production costs (Makkar et al., 2011). Therefore, the current research is focused on the development of production strategies such as culture media formulations using more economic substrates (de Oliveira et al., 2013). The usual optimization method includes changing one parameter at a time while other parameters are kept constant. Compared to the factorial design, this method usually does not show the interaction of parameters, a limitation that can be resolved using statistical methods such as response surface methodology (RSM) (Griffin et al., 1992; Malik and Kerkar, 2019). RSM is a vast statistical method in experimental design that predicts the best culture conditions through the evaluation of factor interactions with a minimal number of experiments (Myers et al., 2016). This statistical method has shown high efficiency in pollutant biodegradation processes and optimization of culture media components for the production of biological compounds, especially biosurfactants (Najafi et al., 2011; Huang et al., 2013; Salar et al., 2016).

Lipopeptide biosurfactants are produced from different *Bacillus* species with carbon sources and have a high potential for use in industry and bioremediation (Das and Mukherjee, 2007; Ghojavand et al., 2008). Therefore, the use of different bacterial species of this genus is highly advantageous for obtaining a valuable and effective bioremediation biosurfactant. Studies show that *Bacillus* spp. strains can use all recyclable sources, especially agricultural-industrial waste, as a carbon source, which further confirms the use of this genus and the related species for bioremediation (Das and Mukherjee, 2007).

This study aimed to evaluate a lipopeptide biosurfactant performance for eliminating heavy metal contamination from soil. In this study, the optimization of the production conditions of lipopeptide biosurfactant by *Bacillus* sp. SHA302 was examined for the first time. We introduced a modified culture medium that both significantly increased the production of lipopeptide biosurfactant and reduced the initial production costs. The biosurfactant production conditions using *Bacillus* sp. SHA302 were optimized with economic carbon sources of sugar beet molasses, potato peel, banana peel, orange peel, date extract, and other growth factors in three statistical analyses of a factorial experiment, Plackett–Burman design, and RSM.

## Materials and Methods

### Soil

The effect of the produced biosurfactant on heavy metal removal from the soil was examined using metal-contaminated soil (Hazrati et al., 2020), for which the physical and chemical characteristics were measured by standard methods (Supplementary Material 1).

### Preparation of Inoculum

The *Bacillus* sp. SHA302 strain was obtained from the microbial collection of the University of Tehran, Iran, and prepared with the method of El-Sheshtawy and Doheim (2014). The strain was cultured at a 10^8^ CFU/ml density in the Bushnell-Haas medium with *N*-hexane (1% *V*/*V*) as a carbon source. The microbial culture medium consisted of ammonium nitrate (1 g/l), potassium dihydrogen phosphate (1 g/l), potassium monohydrogen phosphate (1 g/l), magnesium sulfate (0.2 g/l), iron chloride (0.05 g/l), and calcium chloride (0.02 g/l). The carbon source of the medium was sterilized by a filter (0.22-μm pore size), and the pH of the medium was set to 7.0 using 1 N NaOH before sterilization. The biosurfactant screening tests were done in a prior study (unpublished data).

### Extraction of Biosurfactant and Determination of Critical Micelle Concentration

The biosurfactant was extracted using the method of Sun et al. (2018). After 96 h of incubation, the bacterial suspension was centrifuged (8,000*g*) at 4°C for 20 min. Afterward, 6 N HCl was added to the supernatant to achieve a pH of 2, and the samples were stored at 4°C overnight. Afterward, samples were centrifuged at 8,000*g* for 20 min, and chloroform/methanol (2:1) was added to this solution and the resulting precipitate. Then, the entire process was repeated twice to enhance the extraction. In the end, the organic phase was separated, and the biosurfactant was weighed after evaporation of the solvent.

The critical micelle concentration (CMC) indicates the biosurfactant concentration at which surface tension (ST) reaches its lowest value and then remains constant. It was estimated by plotting the ST vs. biosurfactant concentration (Qiao and Shao, 2010). For this purpose, a stock solution of 1 g/l was prepared from the biosurfactant with sterile distilled water, and then different concentrations of biosurfactant were prepared from this stock solution. Afterward, ST in each concentration was measured in three replications.

### Evaluation of the Biosurfactant Efficiency in the Heavy Metal Release From Soil

The biosurfactant efficiency for soil heavy metal release rate was evaluated with a slightly modified method of da Rocha Junior et al. (2019). Different concentrations of 2 × CMC (230 mg/l), CMC (115 mg/l), and 1.2 × CMC (57.5 mg/l) (25°C, solution volume 50 ml, shaking speed 50 rpm) were made from the biosurfactant, and the pH values of biosurfactant solutions were adjusted to 5.5 ± 0.1. The heavy metal release rate in the treatments was assessed in 1 day. Distilled water was used as a control. To measure the release rate, 5 g of dry soil was added to the soil with 50 ml of the biosurfactant solution (1:10) at different concentrations, and the samples were centrifuged at 5,000*g* for 10 min. The supernatant was discarded, and the soil was dried at room temperature. The heavy metal release rate from the dried soil was measured with an atomic absorption device (Shimadzu, Tokyo, Japan) according to the method of Mulligan et al. (1999). The biosurfactant adsorption rate to the soil was measured according to Chu and Chan (2003).

### The Effects of Salinity, Temperature, and Different pHs on Bacterial Growth

In this study, a range of three salinity factors with different percentages of NaCl (0, 1, 2, 3, 4, and 5%; 30°C; and pH = 7), different temperatures (20, 25, 30, 35, 40, 45, 50, and 55°C and optimum NaCl concentration and pH), and pH values (5.5, 6, 6.5, 7, 7.5, 8, and 8.5 at 30°C) were applied in a nutrient broth medium. The bacterium was inoculated at a concentration of 4% (*V*/*V*) to the medium and incubated in a shaker incubator at 120 rpm for 24 h. Subsequently, optical densities (ODs) were read at 620 nm by a spectrophotometer (JENWAY 6705 UV/Vis), and the growth rate was measured in different conditions mentioned above.

### The First Step of Optimization

#### Selection of Carbon and Nitrogen Sources With a Factorial Experimental Design

The carbon sources of the culture media were orange peel, potato peel, banana peel, date extract, and sugar beet molasses; the nitrogen sources were ammonium ferric citrate, ammonium sulfate, ammonium chloride, potassium nitrate, calcium nitrate, and sodium nitrate. The fruit peels were dried in an oven at 50°C for 4 days and then ground in a mortar to prepare the carbon sources. To prepare stock solutions of carbon, 10% (*W*/*V*) of each carbon source was added to distilled water and autoclaved, and the resulting extract was filtered afterward (Kulkarni et al., 2015). The SHA302 strain was cultured in 100 ml of Bushnell-Haas culture medium in 250-ml flasks with n-hexane along with each of these waste-based materials, including orange peel, potato peel, banana peel, date extract, and sugar beet molasses as carbon sources (1% *V*/*V* and 1% *V*/*V*, respectively) and mineral nitrogen sources (0.1% *W*/*V*) in a pH of 7.0 at 30°C by aeration at 150 rpm. The factorial experiment was carried out with 30 tests in three replicates to determine the best carbon and nitrogen sources in biosurfactant production. Surface tension as a response variable was evaluated in all treatments according to the method of Munguia and Smith (2001). All surface tension values were diluted 10-fold in all three statistical designs, including factorial experiment design, Plackett–Burman, and central composite design (CCD). Afterward, efficient carbon and nitrogen sources as well as other growth factors were designed for bacterial growth using the Plackett–Burman experiment to optimize the biosurfactant production.

### The Second Step of Optimization

#### Design of Nutritional Factors and Culture Conditions With the Plackett–Burman Experiment

After choosing the best carbon and nitrogen sources for biosurfactant production from the first step, the next step of the experimental design was done with the Plackett–Burman method. Ten culture factors of carbon source; nitrogen source; inoculum percentage; salinity percentage; concentrations of iron chloride, magnesium sulfate, potassium monohydrogen phosphate, and potassium dihydrogen phosphate; temperature; and acidity were assessed for biosurfactant production using the Plackett–Burman method. This experiment was designed at two levels to examine *n* - 1 variables with *n* experiments. In this experiment, the lack of factor interactions was assumed as the null hypothesis (Kammoun et al., 2008). In this evaluation, 17 experiments were examined, consisting of 10 main factors, one dummy factor, and five central points; the central points were considered for better experimental error estimation (to make the lack of fit non-significant, five central points were considered), which are interpretable using linear equations demonstrated below.


(1) $$Y=b_{0}+\Sigma b_{i}x_{i}$$

In this equation, *Y* is the response variable (surface tension and quantity of biosurfactant produced), *b*_0_ is the *y*-intercept, and *b*_*i*_ is the effect of the estimated variable. The effect of each variable is calculated using the difference between the average values calculated at the highest (+1) and lowest (−1) levels (Kammoun et al., 2008; Hippolyte et al., 2018). For each factor, a *p*-value of 0.05 was determined as the significant effect of the factor in biosurfactant production. The experiment was designed with the Minitab 16 software.

### The Third Step of Optimization

#### Experimental Design With Response Surface Methodology

Effective factor levels and the interaction of different culture medium components affecting substantial biosurfactant production were analyzed and evaluated with the RSM and the CCD. In this study, the experimental design was composed of 30 experiments with independent variables each at five levels (α+, 1+, 0, 1−, and α−) with six chosen central points for the experiments. Alpha was calculated using the formula α = 2*^k^*^/4^. The number of all experimental components was estimated using the formula *n* = 2^*k* - 1^ + 2*k* + *x*_0_, where *n* is the number of all experiments, *k* is the number of factors being assessed, and *x*_0_ is the number of central points (Dong and Sartaj, 2016; Khoobbakht et al., 2016). Experiments were done in three replicates to find optimal biosurfactant production efficiency, and the obtained data averages were incorporated into a polynomial quadratic regression model.


(2) $$Y=A_{0}+\sum_{i=1}^{n}A_{i}X_{i}+\sum_{i=1}^{n}A_{ii}X_{i}^{2}+\sum_{j=1}^{n}A_{ij}X_{i}X_{j}+\varepsilon$$

In this equation, *Y* shows the estimated response variable (reduction of surface tension and quantity of produced biosurfactant); *A_0_* shows the amount of response in the central point; and *A*_*i*_, *A*_*ii*_, and *A*_*ij*_ show the linear regression, second-order, and interaction terms, respectively. *X*_*i*_ and *X*_*j*_ show orthogonal-coded variables, *n* is the number of dependent variables, and ε shows random error (Montgomery, 2017; Hippolyte et al., 2018). Experiments were designed with the Design Expert Version 12 software.

## Results and Discussion

### The Effect of Lipopeptide Biosurfactant on the Release of Heavy Metals From the Soil

**FIGURE 1:**
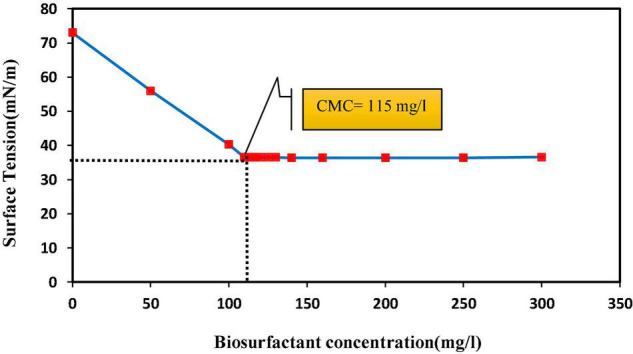
Critical micelle concentration (CMC) of biosurfactant produced by *Bacillus* sp. SHA302 (in Bushnell-Haas culture medium with carbon sources of glucose and n-hexane for 96 h).

**FIGURE 2:**
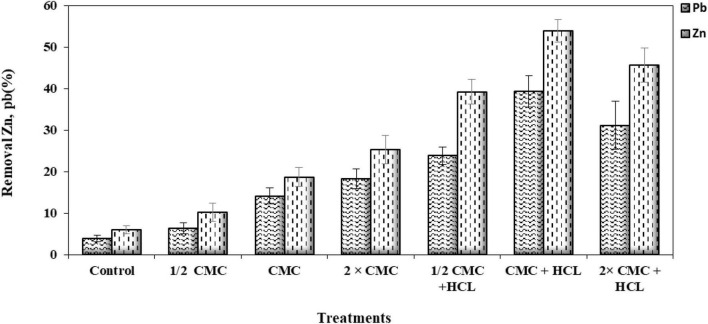
Release of lead (Pb) and zinc (Zn) from contaminated soil at different biosurfactant concentrations in 24 h (HCl, hydrochloric acid; CMC, critical micelle concentration).

It is difficult to predict the efficiency of removing heavy metals from the soil by surfactants, and this is due to the complex and different conditions of the soil system. These factors include soil type, soil contamination intensity, age of contaminants, soil structure, soil solution pH, cation exchange capacity and soil pore size, and surfactant characteristics and concentration, and all are involved in the removal efficiency of surfactant from the soil (Singh and Cameotra, 2004; Xue et al., 2018; Pourfadakari et al., 2019). A critical capability of surfactants is their ability to form micelles (Silva et al., 2014). An increase in biosurfactant concentration lowers ST in the solution, which continues to decrease until the biosurfactant reaches a concentration termed CMC in which micelles are formed (Marchant and Banat, 2012). The results of this study show that from a biosurfactant concentration of 115 mg/l, the ST value was stabilized (36.5 ± 0.6 mN/m) and continued until the end (Figure 1). Many researchers have suggested different CMC values for lipopeptide biosurfactants; for instance, the surface tension of 34 mN/m, CMC of 650 mg/l, and strain *Agrobacterium fabrum* SLAJ731 (Sharma et al., 2019); surface tension of 28–30 mN/m, CMC of about 100 mg/l, and strain *B. megaterium* pL 6 (Pueyo et al., 2009); and surface tension of 32 mN/m, CMC of 350 mg/l, and strain *Bacillus subtilis* ZNI5 (Mnif et al., 2022). The biosurfactant concentration effect on pollutant removal showed that zinc and lead release percentages from soil increased by increasing the biosurfactant concentration. The highest lead and zinc release rates (53.8% ± 1.4 and 39.3% ± 1.7, respectively) were measured in the CMC and acid treatment (Figure 2). With an increase in the biosurfactant concentration, heavy metals in adsorption sites form complexes with the biosurfactant and are further released into the soil solution. Anionic biosurfactants form non-ionic complexes with metallic cations in the soil with higher stability than metallic complexes with soil particles (Franzetti et al., 2014). da Rocha Junior et al. (2019) reported an increase in the removal rate of three metals (lead, zinc, and copper) with increasing the surfactant concentration. The results of Elouzi et al. (2012) in assessing the efficiency of rhamnolipid biosurfactant for the removal of lead and zinc showed an increase in metal removal with increasing the rhamnolipid concentration.

Additionally, the results of this study agree with those of Mulligan et al. (1999) in zinc and copper removal from sediments using a lipopeptide biosurfactant. Doong et al. (1998) presented evidence of an increase in metal removal by increasing the surfactant concentration up to CMC, beyond which a constant trend was seen in the removal of metals. The effective surfactant concentration for the removal of heavy metal pollutants depends on surfactant type and soil conditions (da Rocha Junior et al., 2019). The results of this study on metal removal efficiency by the addition of acid showed an increase in the release rates of both metals up to the CMC concentration and a lowered release rate at the 2 × CMC concentration. This shows that the added acid causes the H^+^ cation to attract to exchange sites and substitute the surface-adsorbed metals, followed by the entry of metals from the adsorption site into the soil solution and their placement within the biosurfactant micelle. At the 2 × CMC concentration, the increased biosurfactant concentrations lead to the competition of the biosurfactant molecules with soil particles for the adsorption of metals and H^+^ cations. Since the hydrated shell of metals is larger than H^+^, they are attached to the exchange sites with less energy than H^+^.

On the other hand, the biosurfactant tends to form a more stable complex, and an increase in its concentration in the soil solution forms a stable surfactant–H complex (McBride, 1994; Bohn et al., 2001). Heavy metals are prone to precipitation at high pH values, which results in lowered pollutant removal and increases metal solubility at a low pH (Yong and Phadungchewit, 1993; Sun et al., 2021). Wu et al. (2015) found that an increase in pH (increased pH-dependent negative charges) reduced the adsorption of a rhamnolipid biosurfactant to the soil, which causes more reactions of the biosurfactant with metals and makes them effective. The lowered metal release rate in the 2 × CMC concentration with acid treatment can be attributed to increased pH-dependent charges with reduced pH and the biosurfactant adsorption to soil with an increase in its concentration, thereby reducing its efficiency. Sarubbo et al. (2018) showed that the biosurfactant produced from *Candida guilliermondii* at a concentration of 0.42% could remove more than 98% of the initial amount of lead and zinc ions.

**FIGURE 3:**
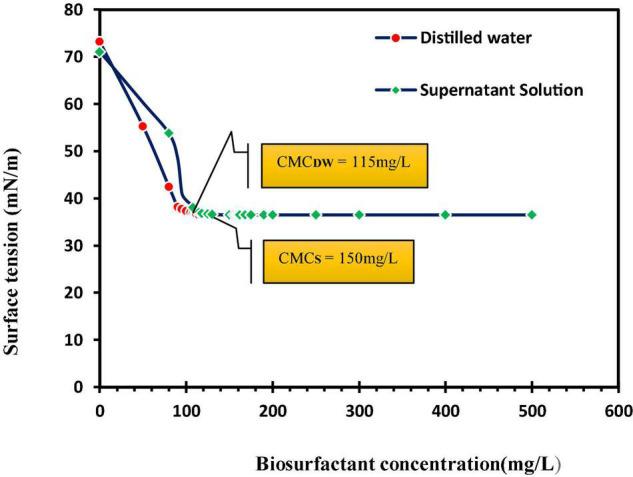
Adsorption of biosurfactant to contaminated soil at different biosurfactant concentrations, CMC_DW_, critical micelle concentration measured in distilled water; CMC_S_, the measured critical micelle concentration of supernatant solutions [supernatant solutions obtained by centrifugation of a mixture of biosurfactants (at different concentrations) and soil].

Grzywaczyk et al. (2021) displayed that soil washing with saponin solution removes 86% zinc and 70% copper. Their results in three different soil types displayed more removal of zinc against copper, and this biosurfactant was considered an effective and environmentally friendly remediation agent. In an investigation on lipopeptide biosurfactant, CMC plus acid treatment had the highest removal of lead (98.7%) and 2 × CMC plus acid treatment had the highest removal of zinc (59.1%) from sandy soil with artificial contamination (Jimoh and Lin, 2020). An important issue concerning surfactant efficiency is their adsorption to the soil. The results of this study show a 23.3% ± 1.2 adsorption of this anionic biosurfactant to the soil (Figure 3). Biosurfactant adsorption to the soil reduces its efficiency in eliminating organic and mineral pollutants (Lee et al., 2000). Biosurfactant adsorption to soil also depends on surfactant characteristics, ionic charge, and soil properties (Calvo et al., 2009).

### Choosing Carbon and Nitrogen Sources

**FIGURE 4:**
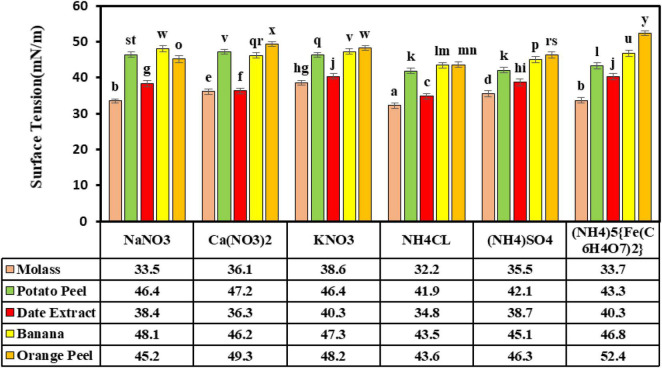
The effects of carbon and nitrogen sources in the production of biosurfactant. Means with the same letters are not significantly different at *P* ≤ 0.05. Error bars indicate the standard deviation (*n* = 3). Duncan test was used for comparisons of means.

The carbon and mineral nitrogen treatments were assessed at a pH of 7.0, 30°C, and an aeration speed of 150 rpm. The results demonstrated that the highest reduction in surface tension (32.2 ± 0.76 mN/m) was obtained in the presence of sugar beet molasses as a carbon source and ammonium chloride as a nitrogen source, which were significantly different from the other nitrogen and carbon sources. The smallest reduction (52.4 ± 0.7 mN/m) in ST was seen with orange peel as a carbon source and the ammonium ferric citrate salt. The results showed that ammonium chloride as a nitrogen source significantly lowered ST alongside three carbon sources of molasses, potato peel, and date syrup (Figure 4). The results of the previous study (data not shown) displayed that the lipopeptide biosurfactant produced in the Bushnell-Haas culture medium with n-hexane and glucose carbon sources had ST and produced biosurfactant values of 36.5 ± 0.23 mN/m and 0.92 ± 0.05 g/l, respectively. Presently, sugar beet molasses is used as a carbon source for biosurfactant production due to its low cost, mineral content, organic compounds, vitamins, and high total sugar content compared to other commonplace carbon sources, such as sucrose and glucose, and is very important for fermentation. Molasses has been reported as an economical carbon source in surfactant production by *Bacillus* spp. (Saimmai et al., 2011). Abdel-Mawgoud et al. (2008) reported a low-cost surfactin production by *B. subtilis* BS5 using an optimized culture medium containing 16% molasses and 5 g/l of NaNO_3_, with a significant increase of 1.12 g/l in surfactin yield. Rane et al. (2017) assessed *B. subtilis* ANR-mediated surfactant production utilizing different carbon sources of glucose, molasses, orange peel, potato peel, banana peel, and bagasse extract. They concluded that 4% molasses carbon source led to the highest biosurfactant production of 0.19 g/l. Joshi et al. (2008) assessed *Bacillus licheniformis* K51, *B. subtilis* 20B, *B. subtilis* R1, and *Bacillus* sp. HS3 for surfactant production. They observed that sugar beet molasses as a carbon source could result in surface tensions of 29.67, 29.33, 30.33, and 30.67 mN/m, respectively. In another study on optimizing the production of lipopeptide biosurfactants produced by *B. subtilis* strain Al-Dhabi-130 with inexpensive sources, molasses was identified as the best source for the production of this biosurfactant (Al-Dhabi et al., 2020).

### Designing Nutritional Factors and Culture Conditions With the Plackett–Burman Design

The screened factors were respectively molasses percentage (*X*_1_), ammonium chloride percentage (*X*_2_), inoculum percentage (*X*_3_), salinity percentage (*X*_4_), iron chloride concentration (*X*_5_), magnesium sulfate (*X*_6_), potassium monohydrogen phosphate concentration (*X*_7_), potassium dihydrogen phosphate concentration (*X*_8_), temperature (*X*_9_), and pH (*X*_10_), which are illustrated along with the three repetition average of ST and the produced biosurfactant in Table 1. Salinity range, temperature, and pH in the Plackett–Burman experiment design were determined using average bacterial growth in different ranges of salinity, temperature, and pH (Supplementary Material 2). The results showed that only four out of the 11 factors, i.e., ammonium chloride concentration, molasses percentage, pH, and magnesium sulfate concentrations, were chosen for further evaluation based on their significance levels (*P*-value < 5%) (Table 2). The significance of these factors means that changes in their values led to the highest effect on changes in ST and biosurfactant production. According to Table 2, the statistical analyses of the models show the significance of the two evaluated statistical models with the regression coefficient percentage (*R*^2^) and adjusted regression coefficient (*R*^2^_adj_) > 80%. Rane et al. (2017) and Hippolyte et al. (2018) considered a regression coefficient of >80% as a threshold for assessing the statistical sufficiency of models. The model coefficients can be calculated using the multivariate regression analysis of responses, ST (*y*_1_), and the extracted biosurfactant (*y*_2_). Negative coefficients in the *y*_1_ model show the positive effect of these factors in biosurfactant production and better efficiency in lowering ST. Additionally, the effect coefficients will be positive if changes in factor levels cause an increase in ST. The results of Eq. 2 demonstrated that factors with high effect coefficients showed bigger effects on ST changes in the culture medium, with the highest effects of ST changes caused by pH (*P* > 0.000, *F* = 646.84), ammonium chloride (*P* > 0.000, *F* = 139.28), magnesium sulfate (*P* > 0.001, *F* = 48.34), and molasses percentage (*P* > 0.008, *F* = 18.49) (except for pH, the other factors had negative effect coefficients). In Eq. 3, the effect coefficients for factors were similar to the *y*_1_ model (except for pH, the other factors had positive effect coefficients). In both the estimated response variables, pH had the greatest effect coefficient. The four factors of pH, ammonium chloride, magnesium sulfate, and molasses percentage were chosen for the RSM to better evaluate and analyze their individual levels. Rane et al. (2017) studied optimum biosurfactant production by *B. subtilis* strain ANR with a Plackett–Burman experimental design. They showed that temperature and molasses were the most significant parameters among factors affecting the production. On the other hand, Almeida et al. (2017) reported that the inoculum percentage and molasses as a carbon source were the most effective factors in surfactant production. In another study on the optimization of lipopeptide biosurfactant produced by *Paenibacillus* D9 (optimal temperature of 30°C, 1.5 ml inoculation, 3% diesel fuel, 4 mM magnesium sulfate, 1% ammonium sulfate, and pH = 7.00), production was reported at 4.11 g/l with a surface tension of 32.2 mN/m (Jimoh and Lin, 2020).

**TABLE 1:** The Plackett–Burman experimental design matrix along with results from surface tension (ST) and produced biosurfactant (BS) as the response variables.

Factors											Response	
Run	*X*_1_ (%)	*X*_2_ (g/l)	*X*_3_ (%)	*X*_4_ (%)	*X*_5_ (g/l)	*X*_6_ (g/l)	*X*_7_ (g/l)	*X*_8_ (g/l)	*X*_9_ (C°)	*X*_10 (–)_	ST (mN/m)	BS (g/l)
1	4	1	2	2	0.02	0.2	1.5	1.5	25	7.0	40.2	1.62
2	8	2	5	2	0.1	0.2	1.5	1.5	25	7.0	31.3	5.55
3	8	1	2	5	0.02	0.2	1.5	1.5	45	7.6	45.4	0.30
4	8	1	5	5	0.1	0.2	1.5	3	45	7.0	39.2	2.12
5	6	1.5	3.5	3.5	0.06	0.3	2.25	2.25	35	7.3	37.1	3.32
6	8	2	2	2	0.02	0.4	3	3	45	7.0	31.5	5.35
7	4	2	2	5	0.1	0.4	3	1.5	45	7.0	35.2	4.34
8	8	1	2	2	0.1	0.4	3	3	25	7.6	47.1	0.10
9	4	2	2	5	0.1	0.2	1.5	3	25	7.6	43.2	1.07
10	6	1.5	3.5	3.5	0.06	0.3	2.25	2.25	35	7.3	36.6	3.82
11	6	1.5	3.5	3.5	0.06	0.3	2.25	2.25	35	7.3	36.8	3.53
12	8	2	5	5	0.02	0.4	3	1.5	25	7.6	44.5	0.74
13	4	1	5	2	0.1	0.4	3	1.5	45	7.6	46.2	0.27
14	4	2	5	2	0.02	0.2	1.5	3	45	7.6	45.1	0.42
15	4	1	5	5	0.02	0.4	3	3	25	7.0	38.5	2.43
16	6	1.5	3.5	3.5	0.06	0.3	2.25	2.25	35	7.3	36.1	4.05
17	6	1.5	3.5	3.5	0.06	0.3	2.25	2.25	35	7.3	37.8	3.07

*X_1_ = molasses, X_2_ = NH_4_CL, X_3_ = inoculum, X_4_ = salinity, X_5_ = FeCL_3_, X_6_ = MgSO_4_, X_7_ = HK_2_PO_4_, X_8_ = H_2_KPO_4_, X_9_ = temperature, X_10_ = pH.*

**TABLE 2:** Analysis of variance (ANOVA) for surface tension (ST) and produced biosurfactant (BS) as the response variables based on Plackett–Burman experiment.

Factors	ST (mN/m)				BS (g/l)			
	DF	Sum of square	*F*-value	*P*-value	DF	Sum of square	*F*-value	*P*-value
**Model**								
*X*_1_	1	7.363	18.49	0.008*	1	1.33	7.15	0.000*
*X*_2_	1	55.47	139.28	0.000*	1	9.434	50.56	0.001*
*X*_3_	1	0.403	1.01	0.360	1	0.1323	0.71	0.438
*X*_4_	1	1.743	4.43	0.089	1	0.4408	2.36	0.185
*X*_5_	1	0.75	1.88	0.288	1	0.5633	3.02	0.143
*X*_6_	1	19.253	48.34	0.001*	1	2.6320	14.11	0.013*
*X*_7_	1	0.163	0.41	0.550	1	0.3888	2.08	0.208
*X*_8_	1	0.27	0.68	0.488	1	0.1496	0.80	0.412
*X*_9_	1	0.403	1.01	0.360	1	0.1408	0.1408	0.75
*X*_10_	1	257.613	646.84	0.000*	1	28.582	153.18	0.000*
Curvature	1	49.28	123.74	0.000	1	8.276	44.35	0.001
Lack of fit	1	0.403	1.02	0.371	1	0.3267	2.16	0.216
*R* ^2^	99.50				98.24			
*R*^2^ adjust	98.39				94.37			

X_1_ = molasses, X_2_ = NH_4_CL, X_3_ = inoculum, X_4_ = salinity, X_5_ = FeCL_3_, X_6_ = MgSO_4_, X_7_ = HK_2_PO_4_, X_8_ = H_2_KPO_4_, X_9_ = temperature, X_10_ = pH (*significant at p < 0.05).


(3) $$Y_{\text{1}}=\text{4}0.\text{617}-0.\text{783}x_{\text{1}}-\text{2}.\text{15}x_{\text{2}}+0.\text{183}x_{\text{3}}+0.\text{383}x_{\text{4}}-0.\text{25}0x_{\text{5}}-\text{1}.\text{267}x_{\text{6}}-0.\text{117}x_{\text{7}}+0.\text{15}0x_{\text{8}}-0.\text{183}x_{\text{9}}+\text{4}.\text{663}x_{\text{1}0}$$

(4) $$Y_{\text{2}}=\text{2}.0\text{27}+0.\text{333}x_{\text{1}}+0.\text{877}x_{\text{2}}-0.\text{1}0\text{5}x_{\text{3}}-0.\text{192}x_{\text{4}}+0.\text{217}x_{\text{5}}+0.\text{468}x_{\text{6}}+0.\text{18}0x_{\text{7}}-0.\text{112}x_{\text{8}}+0.\text{1}0\text{8}x_{\text{9}}-\text{1}.\text{543}x_{\text{1}0}$$

### Experiment Design With the Response Surface Methodology

At this stage, a CCD with six replications in the central point was used to determine the effect of each factor and their interactions on biosurfactant production. The ammonium chloride (*X*_1_), magnesium sulfate (*X*_2_), molasses (*X*_3_), and pH (*X*_4_) factors were assessed as orthogonal variables along with two response variables of ST (*y*_1_) and the produced biosurfactant (*y*_2_). Each parameter in this design was assessed at five levels (−α, +1, 0, −1, and +α), and zero was considered as the central coded value. Optimum amounts in CCD were acquired by solving regression equations and the analysis of results from two- and three-dimensional contour plots. The CCD experimental design matrix is shown in Table 3, and the regression equation analysis of each response variable, along with model coefficients and relevant equations, is demonstrated below.


(5) $$Y_{\text{1}}=\text{32}.\text{87}-\text{1}.\text{35}x_{\text{1}}-\text{1}.\text{3}0x_{\text{2}}-\text{1}.0\text{1}x_{\text{3}}+\text{3}.\text{3}0x_{\text{4}}-\text{1}.\text{41}x_{\text{1}}x_{\text{2}}$$

$$+\text{1}.\text{45}x_{\text{1}}x_{\text{3}}+0.\text{5625}x_{\text{1}}x_{\text{4}}+0.\text{4625}x_{\text{2}}x_{\text{3}}-\text{1}.0\text{5}x_{\text{2}}x_{\text{4}}-0.\text{8375}x_{\text{3}}x_{\text{4}}$$

$$+\text{2}.\text{87}x_{\text{1}}{}^{\text{2}}+\text{1}.\text{51}x_{\text{2}}{}^{\text{2}}-0.0\text{187}x_{\text{3}}{}^{\text{2}}+\text{7}0.\text{62}x_{\text{4}}{}^{\text{2}}$$

(6) $$Y_{\text{2}}=\text{4}.\text{64}+0.\text{4}0\text{75}x_{\text{1}}+0.\text{317}x_{\text{2}}+0.\text{2933}x_{\text{3}}-\text{1}.\text{13}x_{\text{4}}$$

$$+0.\text{4925}x_{\text{1}}x_{\text{2}}-0.\text{4212}x_{\text{1}}x_{\text{3}}-0.\text{2}0\text{62}x_{\text{1}}x_{\text{4}}-0.\text{167}x_{\text{2}}x_{\text{3}}$$

$$+0.\text{295}0x_{\text{2}}x_{\text{4}}+0.\text{2137}x_{\text{3}}x_{\text{4}}-0.\text{8574}x_{\text{1}}{}^{\text{2}}-0.\text{4992}x_{\text{2}}{}^{\text{2}}-0.\text{1119}x_{\text{3}}{}^{\text{2}}-0.\text{3367}x_{\text{4}}{}^{\text{2}}$$

**TABLE 3:** Central composite design (CCD) experiment matrix for two response variables of surface tension (ST) and produced biosurfactant (BS).

Factors					Response	
RUN	X1 (g/l)	X2 (g/l)	X3 (%)	X4 (–)	ST (mN/m)	BS (g/l)
1	1(−1)	0.2(−1)	4(−1)	7.0(−1)	37.1	3.15
2	1(−1)	0.2(−1)	8(+1)	7.0(−1)	34.2	4.26
3	1.5(0)	0.3(0)	6(0)	7.3(0)	33.1	4.82
4	1(−1)	0.4(+1)	8(+1)	7.0(−1)	36.6	2.75
5	2(+1)	0.2(−1)	4(−1)	7.0(−1)	31.7	4.87
6	1.5(0)	0.5(+α)	6(0)	7.3(0)	37.0	3.25
7	1(−1)	0.4(+1)	4(−1)	7.0(−1)	36.2	3.22
8	2(+1)	0.2(−1)	4(−1)	7.6(+1)	45.4	0.6
9	1(−1)	0.2(−1)	4(−1)	7.6(+1)	46.1	0.42
10	1.5(0)	0.3(0)	6(0)	7.3(0)	33.6	4.61
11	1.5(0)	0.3(0)	6(0)	7.3(0)	31.1	4.44
12	2(+1)	0.4(+1)	8(+1)	7.0(−1)	32.2	4.78
13	2(+1)	0.4(+1)	4(−1)	7.6(+1)	34.5	3.65
14	2.5(+α)	0.3(0)	6(0)	7.3(0)	41.5	1.95
15	2(+1)	0.4(+1)	4(−1)	7.0(−1)	30.7	5.35
16	1(−1)	0.4(+1)	4(−1)	7.6(+1)	44.2	0.82
17	1.5(0)	0.3(0)	10(+α)	7.3(0)	29.2	5.74
18	1.5(0)	0.3(0)	6(0)	7.9(+α)	42.0	1.25
19	2(+1)	0.2(−1)	8(+1)	7.0(−1)	35.3	3.52
20	1.5(0)	0.3(0)	6(0)	7.3(0)	33.5	5.15
21	1.5(0)	0.3(0)	6(0)	7.3(0)	33.4	4.52
22	1.5(0)	0.3(0)	6(0)	6.7(−α)	30.2	5.4
23	1.5(0)	0.3(0)	6(0)	7.3(0)	32.5	4.3
24	2(+1)	0.2(−1)	8(+1)	76(+1)	44.8	0.71
25	2(+1)	0.4(+1)	8(+1)	7.6(+1)	37.5	2.52
26	1(−1)	0.2(−1)	8(+1)	7.6(+1)	38.2	2.32
27	1.5(0)	0.3(0)	2(−α)	7.3(0)	36.2	2.65
28	1(−1)	0.4(+1)	8(+1)	7.6(+1)	38.9	2.08
29	1.5(0)	0.1(−α)	6(0)	7.3(0)	41.6	2.1
30	0.5(−α)	0.3(0)	6(0)	7.3(0)	48.1	0.55

*X_1_ = NH_4_CL_4_, X_2_ = MgSO_4_, X_3_ = molasses, X_4_ = pH.*

The results of statistical competence evaluation of models Y_1_ and Y_2_ showed *R*^2^ and *R*^2^-adj values >90%, suggesting that about 90% of independent variable responses can be explained by these models (Table 4). These results corroborate those of Sayyad et al. (2007), who reported that an *R*^2^ of a model closer to 100% better explains the variation between predicted amounts by the model and empirical amounts. According to Table 3, the highest biosurfactant production and the lowest ST are seen in 10% molasses, 1.5 g/l of nitrogen, 0.3 g/l of magnesium sulfate, and a pH of 7.3. In both y_1_ and y_2_ models, the pH with the highest effect coefficient was most effective in both models (Eqs 4, 5). Additionally, Supplementary Material 3 shows the effect of predicted values vs. real model values. In this context, a proximal distribution of observed values to the line is indicative of the proximity of actual values to predicted ones so that *R*^2^ values of 95.08% and 94.26% were calculated for Y_1_ and Y_2_ models, respectively.

**TABLE 4:** Analysis of variance (ANOVA) for the two response variables of surface tension (ST) and produced biosurfactant (BS) based on the central composite design.

	ST (mN/m)				BS (g/l)			
Factors	DF	Sum of square	*F*-value	*P*-value	DF	Sum of square	*F*-value	*P*-value
*X*_1_	1	43.74	17.07	0.0009	1	3.99	13.28	0.0024
*X*_2_	1	40.56	15.83	0.0012	1	2.42	8.06	0.0124
*X*_3_	1	24.40	9.52	0.0075	1	2.07	6.88	0.0192
*X*_4_	1	261.36	101.99	>0.0001	1	30.56	101.79	>0.0001
*X*_1_ *X*_2_	1	31.92	12.46	0.0030	1	3.88	12.93	0.0026
*X*_1_ *X*_3_	1	33.64	13.13	0.0025	1	2.84	9.46	0.0077
*X*_1_ *X*_4_	1	5.06	1.98	0.1802	1	0.6808	2.27	0.1529
*X*_2_ *X*_3_	1	3.42	1.34	0.2659	1	0.4489	1.50	0.2402
*X*_2_ *X*_4_	1	17.64	6.88	0.0192	1	1.39	4.64	0.0479
*X*_3_ *X*_4_	1	11.22	4.38	0.0538	1	0.7310	2.44	0.1395
*X*_1_^2^	1	225.73	88.09	>0.0001	1	20.07	66.86	>0.0001
*X*_2_^2^	1	62.23	24.28	0.0002	1	6.83	22.77	0.0002
*X*_3_^2^	1	0.0096	0.0038	0.9519	1	0.3895	1.30	0.2725
*X*_4_^2^	1	13.68	5.34	0.0355	1	3.11	10.36	0.0057
Lack of fit	10	33.90	3.74	0.0792	10	4.04	4.36	0.0589
Pure error	5	4.53	–	–	5	0.4634	–	–
*R* ^2^	95.08				94.26			
*R*^2^ adjust	90.49				88.91			

*X_1_ = NH_4_CL_4_, X_2_ = MgSO_4_, X_3_ = molasses, X_4_ = pH.*

### Assessment of Variable Interactions in the Response Surface Methodology

**FIGURE 5:**
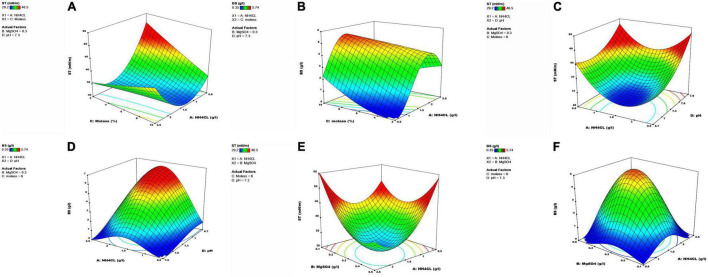
Three-dimensional graphs showing the interactive effects of two independent factors on response variables, while in each graph, the third and fourth variables are set at its center point. **(A)** ST as a function of NH_4_CL and molasses, **(B)** BS as a function of NH_4_CL and molasses, **(C)** ST as a function of pH and NH_4_CL, **(D)** BS as a function of pH and NH_4_CL, **(E)** ST as a function of MgSO_4_ and NH_4_CL, **(F)** BS as a function of MgSO_4_ and NH_4_CL.

The results clearly demonstrated that magnesium sulfate and ammonium chloride concentrations, molasses percentage, and pH significantly affected the reductions of ST and the produced level of the biosurfactant. The interactions of these variables with response values were assessed using three-dimensional plots against both response variables. The results showed that ST initially decreased and then increased at a low molasses concentration (2%) with an increase in ammonium chloride level. The same trend was also seen at higher molasses concentrations, but the intensity of ST changes was higher at low quantities of molasses than at higher levels (Figure 5A). The lowest ST amount (1.5 g/l) was seen in 10% molasses and the central point of ammonium chloride, and ST increased by increasing the distance from the central point of ammonium chloride. According to Figure 5B, an increase in molasses percentage increased the produced biosurfactant level, and ammonium chloride concentration in the central point had the highest effect on increasing biosurfactant production. Therefore, the medial quantity of ammonium chloride (1.5 g/l) and the highest molasses percentage (10%) had the most significant effect on biosurfactant production.

A nitrogen source is one of the critical factors in biosurfactant production (Burkovski, 2003). Nitrogen is a nutrient that plays an essential role in cellular metabolism. A decrease in nitrogen content in the culture medium interrupts protein production and shifts cellular metabolism to carbohydrate production, thereby increasing biosurfactant production. The presence of high amounts of nitrogen sources in the culture medium lowers biosurfactant production and directs metabolism toward cellular growth (Abalos et al., 2002). This study observed a reduction in biosurfactant production by increasing ammonium chloride concentrations above the central point concentration. High concentrations of salt and minerals in molasses lower biosurfactant production and activity due to an increase in osmotic pressure; therefore, it reduces cell viability and ceases biosurfactant production (Doelle and Doelle, 1990). Wei et al. (2005) showed that biosurfactant production increased initially with an increase in the molasses concentration until it reached a maximum, and the production became stable or decreased afterward. During the optimization of the *Candida tropicalis* strain, an increase in molasses percentage resulted in a significantly reduced surface tension (Almeida et al., 2017). Similarly, our results showed that an increase in molasses percentage increased the biosurfactant production level, which can be due to the halophilic property of the bacterium, for which salinity may not be a growth-limiting factor at a high molasses concentration (refer to Supplementary Material 2a).

At low ammonium chloride quantities, ST rose with increasing pH, and this increase was more pronounced at lower ammonium chloride concentrations (Figure 5C). In low pH values, an increase in ammonium chloride first led to an increase in biosurfactant production, but it decreased at concentrations higher than the central point of ammonium chloride. The highest biosurfactant production occurred at the central point concentration of ammonium chloride, and changing intensity was higher at low than at high pHs (Figure 5D). One of the important characteristics of most organisms is their strong dependency on pH conditions for growth and secondary metabolite production (Najafi et al., 2011). The SHA302 strain could grow in a pH range of 5.0–9.0, with the highest performance for biosurfactant production in pH 7.3. Since microorganisms grow well at a pH of 7, biosurfactant production is less affected at lower pHs than at higher pHs, which significantly reduces the production. The results of this study showed that the SHA302 strain had the highest growth at a pH of 7.5 (refer to Supplementary Material 2c), and biosurfactant production decreased with an increase in pH. As demonstrated in Figure 5E, ST initially decreased at low ammonium chloride quantities and then increased by increasing magnesium sulfate levels. ST showed a decrease at high ammonium chloride concentrations. As shown in Figure 5F, different ammonium chloride quantities and an increase in magnesium sulfate resulted in an initial increase in the biosurfactant production and then began to decrease with the highest production at the central point concentration (NHCl = 1.5 g/l, MgSO_4_ = 0.3 g/l) for both factors.

### Finding Optimum Conditions for Biosurfactant Production

**FIGURE 6:**
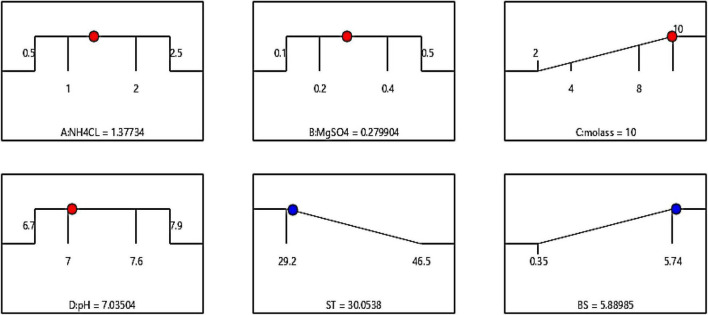
Optimal conditions predicted by the software, by adjusting the molasses factor levels to the maximum value (10%).

The optimum conditions for both models (surface tension/biosurfactant production) are shown in Figure 6. Independent input variables can be adjusted to their maximum, minimum, or any target point, while the response variable is usually set on minimum or maximum values. The software adjusted these conditions by placing molasses at the highest concentration (10%) and the three factors of ammonium chloride, pH, and magnesium sulfate on predetermined levels of 1–2 g/l, 7–7.6, and 0.2–0.4 g/l, respectively. The results showed that by placing molasses at a maximum and the other factors in the determined range (Figure 3), the surface tension and the amount of extracted biosurfactant were 30.08 mN/m and 5.89 g/l, respectively. To compare the accuracy of the estimated response values by the software with the actual values, ST and the produced biosurfactant (BS) were measured at the amounts suggested by the software. The obtained amounts were 30.48 mN/m and 5.76 g/l, respectively, indicating the high precision of the production optimization model with the values obtained from the experiment.

### Economic Challenges and New Production Approaches

The global market for biosurfactants is growing by 5.6% from 2017 to 2022 and may reach 5.52 billion dollars (Gaur and Manickam, 2021). Considering the increasing interest in the use of biosurfactants in recent decades, the market for the production of these compounds is fundamentally missing because of the lack of access to cheap raw materials and economic issues related to production (Helmy et al., 2011). The cost of producing biosurfactants commonly relies upon the volume of the bioreactor, the cost of the raw materials, and the cost of separating them. In the production process of biosurfactants, the rate of biosurfactant foaming, the volume of the bioreactor, and the biosurfactant’s viscosity can have harmful effects on its production (Dolman et al., 2019). Promising strategies for increasing the production of biosurfactants in the last decade have been used to overcome the economic challenges of its production, some of which are as follows: the use of growth stimulants (Roelants et al., 2016), application of nanoparticles (Kiran et al., 2014), production of biosurfactants simultaneously with other biological compounds (Kavuthodi et al., 2015), the use of immobilized microorganisms (Heyd et al., 2011) and, so on. These strategies can be cost-effective in reducing the cost of producing biosurfactants. Therefore, taking these strategies more seriously is an essential step towards increasing productivity, making the production process more economical, as well as resolving concerns about solid waste disposals by converting them efficiently into low-cost industrial solids. Such solutions make agricultural waste valuable and profitable products (Singh et al., 2019).

## Conclusion

This research aimed at using biosurfactants in the release of heavy metals and finding optimum conditions for biosurfactant production with the *Bacillus sp*. SHA302 strain using low-cost feedstock. The *Bacillus* sp. strain SHA302, with 93.98% phylogenetic similarity to registered strains in GenBank, is the first report in biosurfactant production, and a strain with this low percentage of similarity is most likely novel. In this study, a new culture medium was formulated to produce lipopeptide biosurfactant, resulting in increased productivity, reduced costs, and commercialization in agricultural industries. The cost reduction results displayed that to produce 1 L of formulated medium and 1 g of low-purity biosurfactant, 0.13-dollar and 2.1-dollar costs are required, which are pretty economical quantities to produce. Fortunately, with a meager purification rate, this biosurfactant showed outstanding efficiency in agriculture, and this issue played an essential role in reducing production costs. Our previous study reported the high efficiency of this biosurfactant in releasing oil pollutants from soil, and this study demonstrated its ability in heavy metal removal from the soil. Therefore, the optimization of this biosurfactant can be an effective solution for treating saline-sodic soils with long-term contamination (both petroleum hydrocarbons and heavy metals).

## Data Availability Statement

The original contributions presented in the study are included in the article/Supplementary Material, further inquiries can be directed to the corresponding author.

## Author Contributions

SK: conception and design of the study, analysis and/or interpretation of data, writing and drafting the manuscript, and revising the manuscript critically for the final version. HG: writing and drafting the manuscript and revising the manuscript critically for the final version. AP: conception and design of the study, analysis and/or interpretation of data, and revising the manuscript critically for the final version. HA: interpretation of soil analysis. All authors contributed to the article and approved the submitted version.

## Conflict of Interest

The authors declare that the research was conducted in the absence of any commercial or financial relationships that could be construed as a potential conflict of interest.

## Publisher’s Note

All claims expressed in this article are solely those of the authors and do not necessarily represent those of their affiliated organizations, or those of the publisher, the editors and the reviewers. Any product that may be evaluated in this article, or claim that may be made by its manufacturer, is not guaranteed or endorsed by the publisher.
