# Supplementary material for: Formulation of a Culture Medium to Optimize the Production of Lipopeptide Biosurfactant by a New Isolate of Bacillus sp.: A Soil Heavy Metal Mitigation Approach
Source: Front Microbiol. 2022 Mar 8;13:785985. doi: 10.3389/fmicb.2022.785985 (PMC8979173; doi:10.3389/fmicb.2022.785985)
Supplement: Supplementary file 1 [file Data_Sheet_1.docx]

### Supplementary Materials

### Sup. 1. Soil properties used in this study

| Term | Amount |
| --- | --- |
| Soil texture | Clay Loam |
| pH | 7.43±0.03 |
| EC (μs/cm) | 221.6±4.7 |
| SOC (g/kg) | 11.44±0.78 |
| Available Nitrogen (mg/kg) | 19.5±1.5 |
| Available Phosphorus (mg/kg) | 13.2±0.4 |
| Exchangeable K (mg/kg) | 487.78±11.15 |
| Exchangeable Na (mg/kg) | 38.0±4.0 |
| Zn(mg/kg) | 2303±139 |
| Pb(mg/kg) | 1262±37 |

Sup. 2. Growth rate of SHA302 strain at (a); different salinity, (b): temperatures and, (c): pH at 620 nm


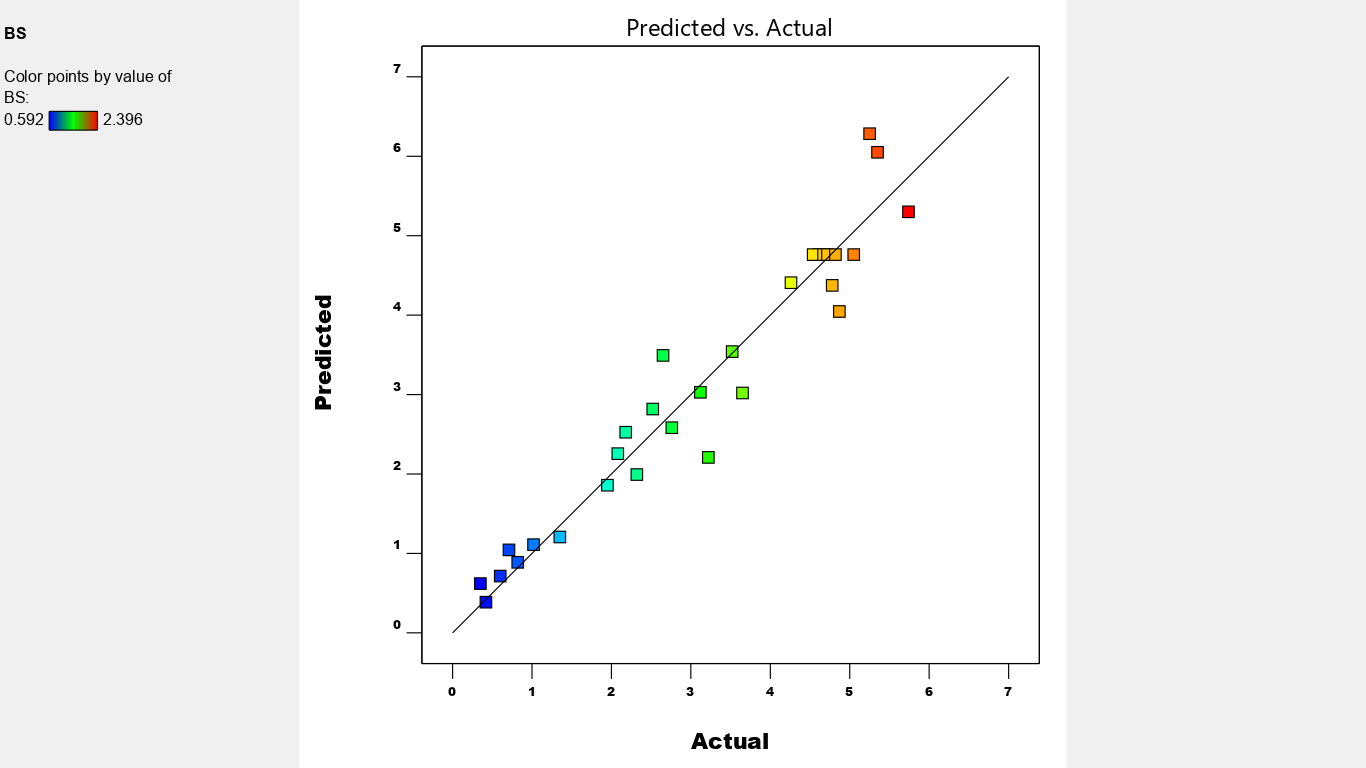
 **
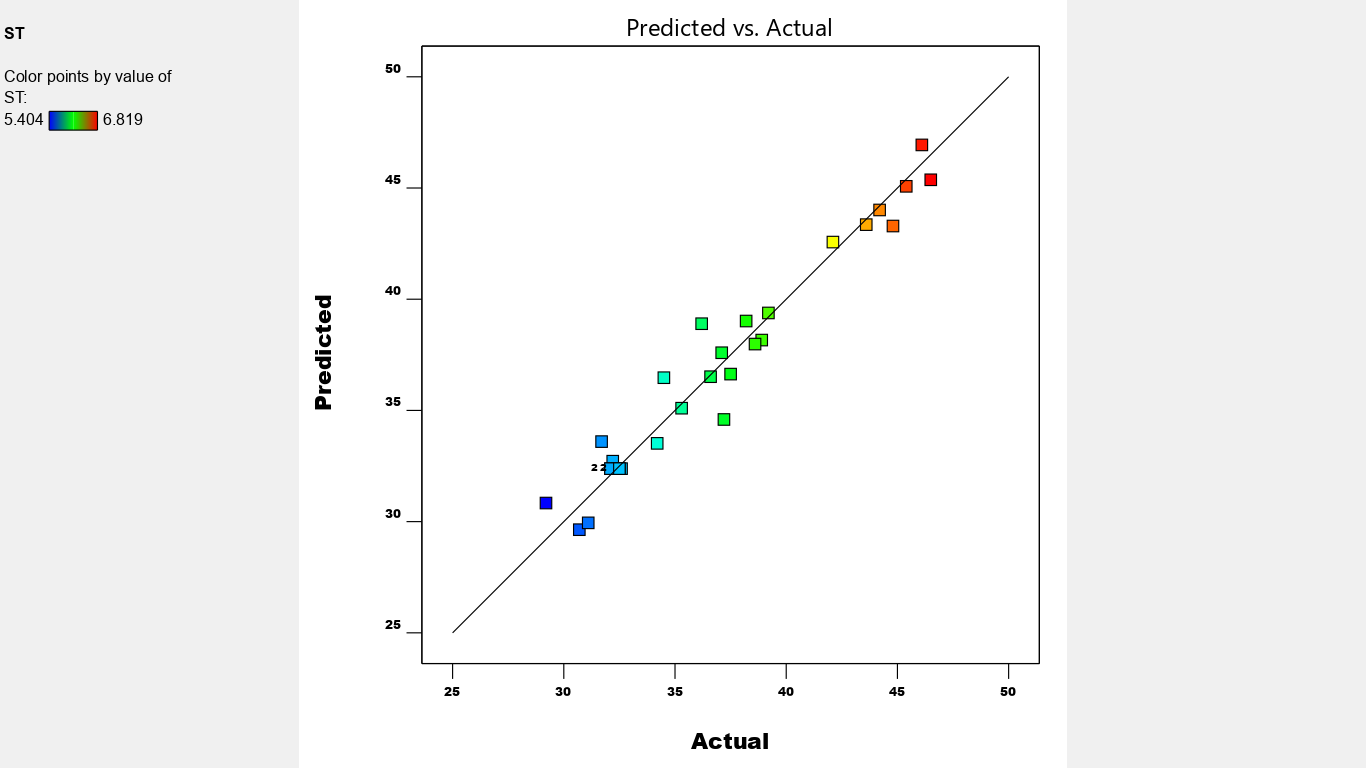
**

Sup. 3. Predicted values versus the actual values of the two response variables (a); Surface Tension, (b): produced biosurfactant from SHA302 strain.
